# Conceptual Piezoelectric-Based Energy Harvester from In Vivo Heartbeats’ Cyclic Kinetic Motion for Leadless Intracardiac Pacemakers

**DOI:** 10.3390/mi15091133

**Published:** 2024-09-06

**Authors:** Majid Khazaee, Sam Riahi, Alireza Rezania

**Affiliations:** 1 Department of AAU Energy, Aalborg University, 9220 Aalborg East, Denmark; mad@energy.aau.dk; 2 Department of Cardiology, Aalborg University Hospital, 9000 Aalborg, Denmark; sar@rn.dk; 3 Department of Clinical Medicine, Aalborg University, 9000 Aalborg, Denmark

**Keywords:** energy harvesting, leadless pacemaker, piezoelectric, heart motion

## Abstract

This paper studies the development of piezoelectric energy harvesting for self-powered leadless intracardiac pacemakers. The energy harvester fit inside the battery compartment, assuming that the energy harvester would replace the battery with a smaller rechargeable battery capacity. The power output analysis was derived from the three-dimensional finite element analysis and in vivo heart measurements. A Doppler laser at the anterior basal in the right ventricle directly measured the heart’s kinetic motion. Piezoceramics in the cantilevered configuration were studied. The heart motion was periodic but not harmonic and shock-based. This study found that energy can be harvested by applying periodic bio-movements (cardiac motion). The results also showed that the energy harvester can generate 1.1 V voltage. The effect of various geometrical parameters on power generation was studied. This approach offers potential for self-powered implantable medical devices, with the harvested energy used to power devices such as pacemakers.

## 1. Introduction

Cardiac pacemakers [1] can help prevent some of the 17.9 million annual deaths caused by cardiovascular diseases [2]. Conventional pacemakers, implanted in the chest and connected to the heart by leads, consist of an automatic generator, wires with electrodes, and a battery. However, these pacemakers have drawbacks such as lead dislodgement, short lifespan, and infection risk. On the contrary, intracardiac leadless pacemakers (ICLPs) are more miniature [3] and do not require leads, which eliminates many of the complications associated with conventional pacemakers [4] and also decreases the risk of infections [5]. However, ICLPs have their limitations, such as shorter battery life (approximately 10 years [6]) and the inability to retrieve them once these devices are implanted [7,8]. Further research is needed to develop novel self-powering ICLPs to overcome these limitations. Multiple ICLPs have been implanted inside animal hearts [9]. However, multiple ICLPs have not been implanted in humans, and inserting multiple ICLPs in human hearts is challenging. Additionally, the psychological fear of having a low battery level [10] and the long-term non-removability of ICLPs may limit their use, especially in younger patients [11].

The human heart produces a significant amount of kinetic energy, and harvesting this energy with high-performance piezoelectric (PE) materials could spur a medical paradigm shift. The only ICLP available on the market, Micra (Medtronic, Minneapolis, MN, USA), consumes a small amount of power (2 µW power [12]) and can be self-powered if a part of the heart kinetic power (0.93 W [13]) is harvested as electricity. Current energy-scavenging technologies have limitations; for example, solar power [14] cannot be used inside the body, electromagnetic [15] power cannot be used for great depth, triboelectricity [16] has a short lifetime, biofuel cells [17] have low power and are not genetic, and thermoelectric generators [18] can be heavy or ineffective at low body temperature differences. Furthermore, these technologies can have a high infection risk [19]. PE ceramics are strain-sensitive with high PE coefficients. The PE effect can be created by applying mechanical stress on PE materials, which generates an electrical voltage. Periodical bio-movements were used for PE energy, blood flow [20], tissue contraction [21], and cardiac/lung motions [19,22].

The frequency of cardiac motion is relatively low (1–3 Hz). There are other challenges for heart kinetic energy harvesting, one of which is the precise measurement of the heart motion since the heart muscles do not have sinusoidal vibration and consist of a low-duty cycle [23]. A linear zigzag structure and nonlinear magnetic coupled cantilever beam were investigated for the leadless pacemaker study [24]; however, the size of these considered structures did not fit into the available leadless pacemaker. The flexible cylinder of a leadless pacemaker under blood flow excitation has been investigated [25]; however, the tissue around this flexible structure over time will make this energy harvester ineffective. Jackson et al. [23] introduced two aluminum nitride-based MEMS rectangular cantilever beams and analyzed these beams using the simplified analytical vibration equation. The tailored curved edge cantilever MEMS beam was also investigated [26].

As a mature technology, piezoelectricity generates energy from the cyclic mechanical strain. Therefore, the motion frequency and the strain magnitude are two factors that play an influential role. Macro-scale PEHs must be heavy and large (>25 g and >20 mm diameter) for the required power [27]. A central vein of research is focused on piezoelectric new materials or and the development of materials [28,29,30], including electrode design for piezoelectric nanogenerators [31], piezoelectric nanowires [32], piezoelectric β-programming composites [33,34], lead-free piezoelectric components [35,36], and flexible thin-film materials [36,37,38]. Stretchable thin films [36,39], three-dimensional printing of tailoring anisotropic piezoelectricity [40], stress rearrangement for wearables [41], and piezoelectric bond engineering [42] have been investigated to improve piezoelectric energy generation. Moreover, the base material for these studies [37,39,43,44] is a polymer-based piezoelectric material with low piezoelectricity [45]. The heart, with its complex geometry and structure, moves differently in various regions [46]. It has been shown that the location and orientation of a flexible piezoelectric patch on the heart’s external surface can alter the amount of piezoelectric energy generated [47].

**Figure 1:**
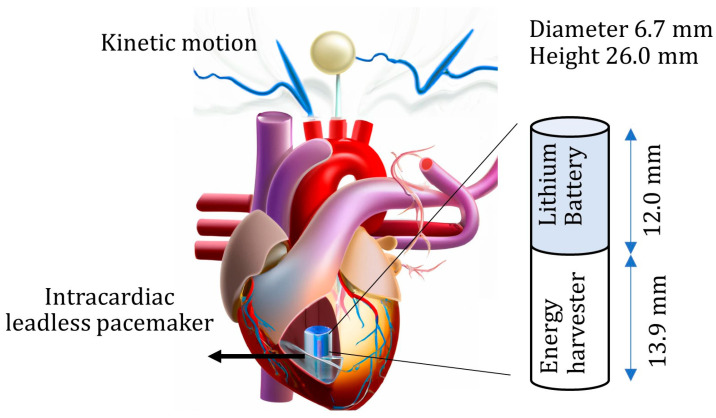
Intracardiac leadless pacemaker and the heart kinetic-energy-based harvesting concept.

This paper aims to assess the harvesting of the heart’s kinetic energy for leadless pacemakers through a conceptual approach for integrating PE energy harvesters into commercial leadless pacemakers, as shown in Figure 1. First, a laser sensor directly measures the heart’s precise kinetic motion during animal surgery. Second, the power output from piezoelectric materials is investigated using a small-volume PE energy harvester. The heart motion results act as a guide for dynamic analysis of piezoelectricity. This study aims to a open up a new avenue of research with real-time heart signals in energy-harvesting studies, which is novel in transient piezoelectric real-time heart analysis. The energy generator results indicate satisfactory energy harvesting for advanced self-powered leadless pacemakers.

## 2. Heart Kinetic Motion

The human heart’s kinetic motion, amplitude, and motion shape can present valuable data for designing energy harvesters; however, precise data on the heart’s motion type and amplitude are challenging. The heart is a complex organ having both electrical and mechanical indexes. The electrocardiogram (ECG), a common diagnostic tool, is a measure of the electrical activity of the heart. The mechanical kinetic motion of the heart, which refers to the movement of the heart muscles, can provide valuable information about heart function. Electrical and dynamic heart motions show similarities and characteristics as a complete analysis of heart activities. ECG signals are employed from the literature, but since data on the heart’s dynamic motion are limited, animal surgery tests on a living pig’s heart were carried out.

**Figure 2:**
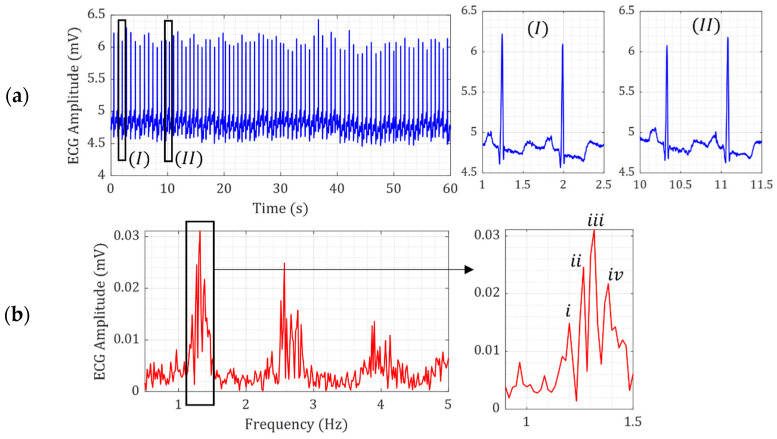
(**a**) ECG signal from a 69-year-old male patient with normal sinus rhythm [27] with zoomed-in views at two 1.5 s time intervals (I) and (II) are zoomed-in ECG signals, and (**b**) Fourier transform of the ECG and (i–iv) zoomed-in view of typical heart rate frequencies.

ECG open datasets are widely available [48]. Figure 2a,b show the time and frequency domain signals of ECG measurement of a 69-year-old male volunteer [48]. The ECG signal over 60 s for one patient shows that the amplitude of ECG is slightly variable, as shown in Figure 2a. This variation is further illustrated in the zoomed-in views of the ECG signals in Figure 2a-I and Figure 2a-II. Nevertheless, the similarity of all ECG signals is the shock pattern in the ECG signal, demonstrating the sudden motion of the heart in a short period. The frequency-domain ECG signals in Figure 2b show that the heart rate changes slightly between 1.2 Hz and 1.38 Hz, even for 60 s.

For the measurement of mechanical heart muscle movement, the animal surgery measurements were carried out for in vivo kinetic heart motion using a Doppler laser displacement meter, type ILD1320-50mm (MICRO-EPSION, Ortenburg, Germany), measuring the displacement at a sampling rate of 2000 Hz. A fixed-based frame kept the laser facing down to the middle line of a beating female pig’s heart. Animal treatment was carried out according to Danish Animal Experiments Inspectorate’s requirements, and this institution approved the study (no. 2021-15-0201-0082). The animal was a 35 kg female pig with one week of acclimation.

**Figure 3:**
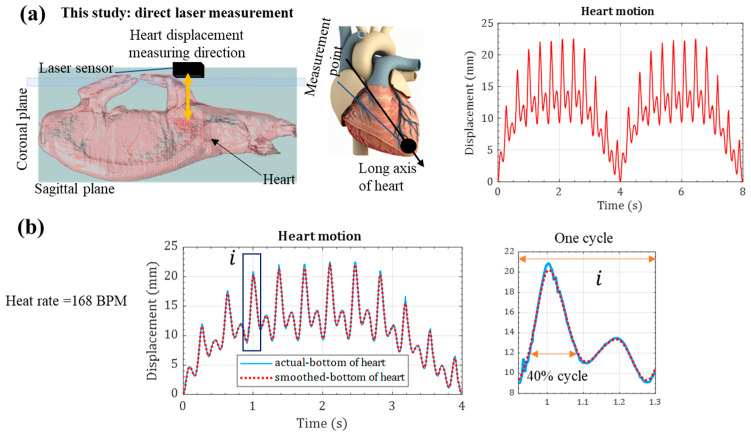
Kinetic heart motion during heart beating and respiration: (**a**) direct laser measurement of the lower point of the heart in the midline, and (**b**) zoomed-in shock-based cardiac cycle motion.

Direct heart measurement was implemented in the sagittal plane during open heart surgery (Figure 3a). The pig’s heart rate was ~160 beats per minute (BPM) during the animal test due to atrial fibrillation. Figure 3a shows the heart measuring point in the coronal plane. The heart displacement during 11 cardiac cycles of 1660 BPM is demonstrated in Figure 3a. Like the ECG signal, direct laser displacement shows a high-amplitude short-duty cycle half-wave motion and a low-amplitude sinusoidal motion. Breathing also created a ~0.25 Hz frequency motion of the heart, as shown in Figure 3a. Therefore, the heart’s kinetic motion consisted of respiratory and heart-beating motions. The maximum heart movement for the cycles was ~22 mm. However, excluding the respiratory motion, the heart motion caused by the heart beating was only ~12.8 mm. A smoothening version of these cycles was used [49] for computer simulation of energy harvesters (Figure 3a), as transient solutions require a smooth input function. These displacement measurements were in the Z-axis. Due to the limitation of in vivo tests, measuring the X and Y axis displacements was impossible when using a remote laser displacement sensor.

For comparison, we kinetic heart measurements were carried out using an endoscopic monocular vision system of a 25 kg porcine model in accordance with the work of Sauvee et al. [50]. Respiratory-induced heart and heart-beating motions were visible in this study as well. Nevertheless, the Sauvee heart-beating movement was not as uniform as the direct measurement in this study’s animal testing. Therefore, these presented laser measurements provided valuable inputs for a dynamic analysis of the heart, especially for energy-harvesting technologies. Moreover, significant shock-based amplitude heart movement can be seen in Figure 3a, which lasted approximately 40% of the cardiac cycle. The shock-based motion was like the ECG signal; however, the duration of the dynamic movement was greater than that of the ECG signal.

**Figure 4:**
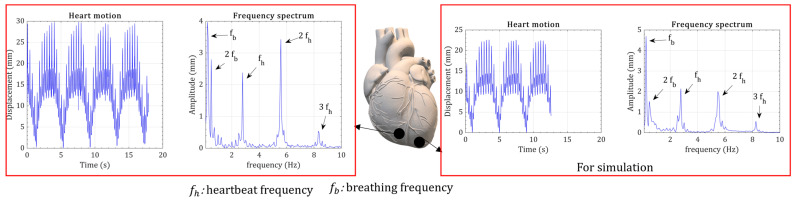
The measurements of in vivo heart displacement at two points on the heart surface.

The ECG shows many small fluctuations in the signal compared to the mechanically measured heart motion. The ECG signal presents valuable information about the heart’s electrical function, even though the limitation is the missing point between the electrical function of the heart and the mechanical motion. Therefore, the heart’s kinetic motion is essential for energy-harvesting applications. The heart’s kinetic motion can be different depending on the regions in the heart. Two in vivo measurements are shown in Figure 4, where the measurement point is around the leadless pacemaker placement. Breathing and heartbeat frequencies can be seen in both frequency spectrums. Breathing ($f_{b}$) and heartbeat ($f_{h}$) frequencies can be seen in both frequency spectrums. The higher harmonics of breathing and heartbeat frequencies can be seen in the frequency spectrum. The third harmonic of breathing is negligible, and the third harmonic of heartbeat motion has the smallest peak. The amplitude of heart measurements was slightly different. For the simulations, the heart measurement with the lowest amplitude was selected.

## 3. Results and Discussion

A cantilevered energy harvester was studied, which is the most widely used boundary condition in energy harvesting [49]. The cantilevered energy harvester was a bimorph with two PZT-5A piezoelectric layers (PIEZO.COM, Woburn, MA, USA), a titanium center shim, and an added tip mass. The outer cylindrical shell of the leadless pacemakers was made of titanium, which was moisture-resistant and biocompatible with the tissues. Thus, using lead-based ceramic piezoelectric material was not critical; however, the recent advances in lead-free materials have indicated that high-performance lead-free materials may be alternatives [51]. Studies were carried out by finite element analysis using COMSOL Multiphysics version 6.1, with transient solutions with the real-time heart displacement measurements in Section 2.

### 3.1. Verification of Model

**Figure 5:**
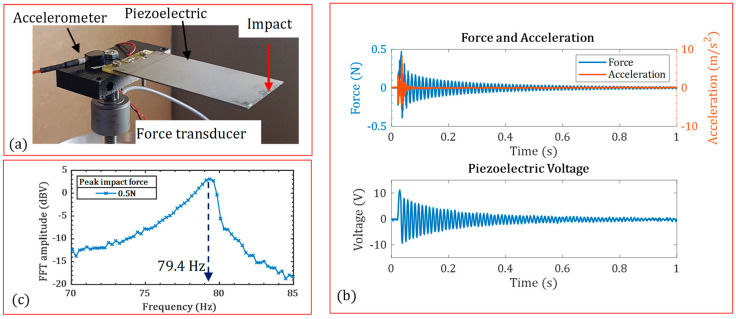
(**a**) The experimental setup; (**b**) measured impact force, acceleration, and piezoelectric voltage under the impact; and (**c**) fast Fourier transform of voltage.

The COMSOL finite element (FE) model was verified against the experimental data. A bimorph piezoelectric energy harvester was subjected to impact load. A controllable impact hammer created the impact load. The B&K 4517 accelerometer (HBK, Virum Denmark), 10.51 mV/g, and B&K 8230 force transducer (HBK, Virum, Denmark), 22.1 mV/N, measured the responses. An oscilloscope carried out the piezoelectric response and spectrum analysis. Figure 5a shows the experimental setup. The impact’s force, acceleration and piezoelectric voltage are shown in Figure 5b. The fast Fourier transform (FFT) of piezoelectric voltage was calculated at 79.4 Hz as the first natural frequency of the piezoelectric structure.

Table 1 shows the characteristics and material properties of the tested piezoelectric harvester. The presented COMSOL finite element identified the first natural frequency with a 2.7% error, see Table 2. Higher harmonics were not identified between 1–500 Hz in the experimental tests. This agreement verifies the COMSOL model, which will be used later in the energy harvester assessment of leadless pacemakers.

### 3.2. Energy Harvesters for Leadless Pacemakers

**Figure 6:**
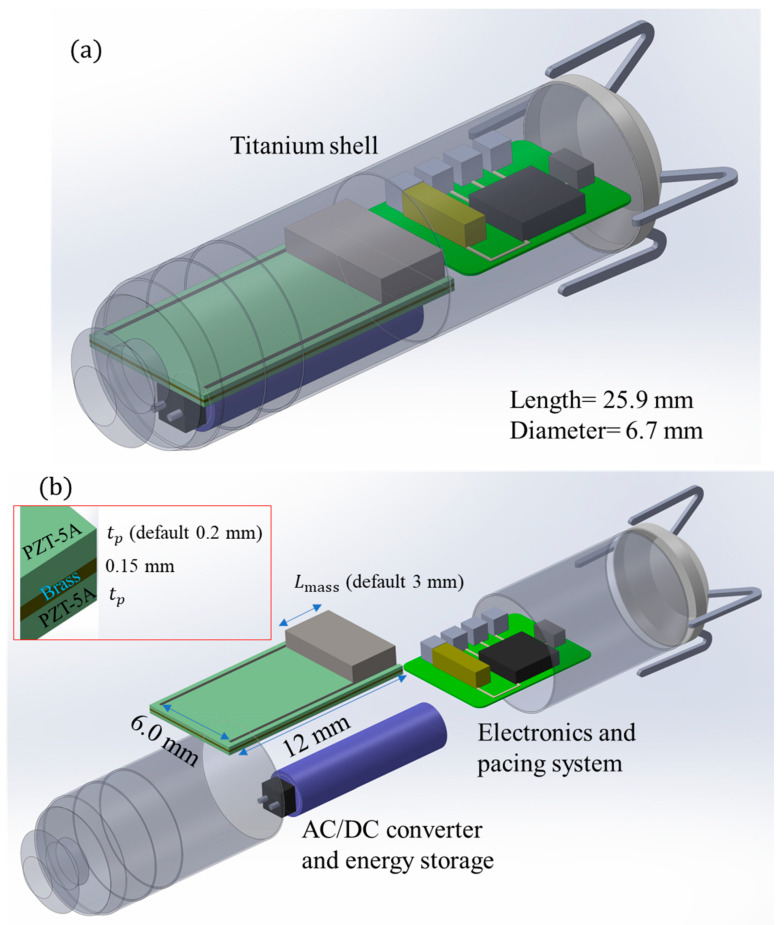
(**a**) Intracardiac leadless pacemaker: concept of an energy-harvesting beam, rectifier, and energy storage; (**b**) dimensions of the energy harvester and study parameters.

An intracardial leadless pacemaker was conceptually modified so that an energy harvester replaced the main bulky battery, see Figure 6a. The dimensions of the energy harvester are shown in Figure 6b. The ECG signals demonstrate that a patient’s heart rate can vary slightly. The output wires of the piezoelectric beam shall be connected to an AC/DC converter, which is then fed to an energy storage component. This energy storage component will provide the power for the pacing system. Electricity storage is the next level of conceptual development.

The heart’s kinetic motion was measured during animal open heart surgery, and the performance of a piezoelectric harvester was evaluated. First, through transient analysis, we assessed the mechanical deformation under heart motion. The heart motion was the in vivo measured heart signal, including respiration and heart beating motions. A time period of 4 s was used for simulation, which includes one respiration cycle and ten cardiac cycles.

**Figure 7:**
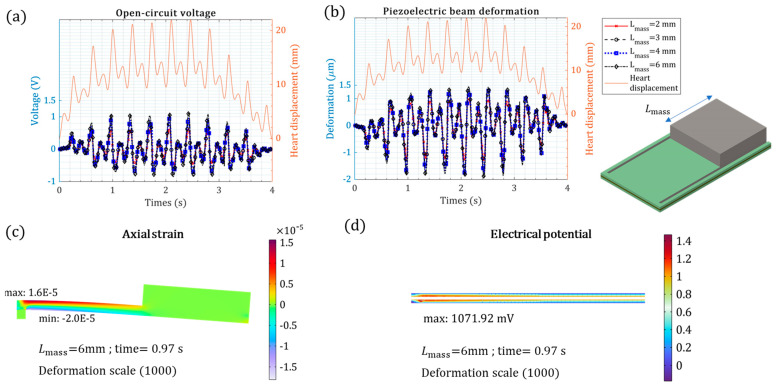
The piezoelectric cantilevered beam mechanical response under heart kinetic motion versus different tip masses: (**a**) open-circuit piezoelectric voltage; (**b**) piezoelectric beam tip deformation; (**c**) axial strain for t = 0.97 s, where the voltage is maximum; (**d**) surface electrical potential for t = 0.97 s, where the voltage is maximum.

Figure 7 shows the mechanical and electrical performances of the cantilevered beam under the heart input kinetic motion when the tip mass varies. The heart’s kinetic motion is periodic. The piezoelectric voltage generation pattern followed the cardiac kinetic motion, as shown in Figure 7a,b. The breathing motion of the heart had a negligible effect on the output voltage, which might be due to the ultralow frequency. The piezoelectric energy harvester is a cantilevered beam with a tip mass experiencing base excitation. This setup was the same for the COMSOL Multiphysics models. The open-circuit voltage was approximately 1.0 V for $L_{\mathrm{mass}}$ = 6 mm. Adding tip mass increased the beam deflection (Figure 7a) and thus output voltage (Figure 7b). The range of strain varied from 8 µε (ε: strain) for $L_{\mathrm{mass}}$ = 2 mm to 16 µε (ε: strain) for $L_{\mathrm{mass}}$ = 6 mm. Even with a highest tip mass weight of $L_{\mathrm{mass}}$ = 6 mm, the von Mises stress was ~1.7 MPa, which is very small because the input acceleration from the heart is not tremendous. The maximum strain was minor compared to the material’s strength; therefore, applying a tip mass was safe.

**Figure 8:**
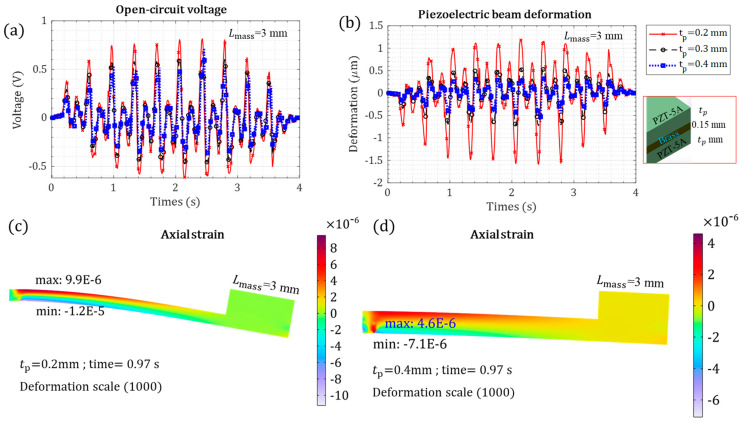
The piezoelectric cantilevered beam mechanical response under heart kinetic motion versus different piezoelectric layer thicknesses: (**a**) open-circuit piezoelectric voltage; (**b**) piezoelectric beam tip deformation; (**c**) axial strain with $t_{p}$ = 0.2 mm for t = 0.97 s, where the voltage is maximum; and (**d**) axial strain with $t_{p}$ = 0.4 mm for t = 0.97 s, where the voltage is maximum.

The mechanical and electrical performance, affected by the piezoelectric-layer thickness, was studied, as shown in Figure 8. The thicker piezoelectric layer caused lower voltage generation (Figure 8a), as increasing the thickness increased the energy harvester stiffness and reduced the mechanical train and tip deformation (Figure 8b). The 0.2 mm piezoelectric layer generated double the voltage that the 0.4 mm piezoelectric layer did, as shown in Figure 8c,d. The higher voltage is due to the higher strain axial in the thin piezoelectric layer. A thinner piezoelectric layer would be even more beneficial, because the energy harvester mass would be lower.

**Figure 9:**
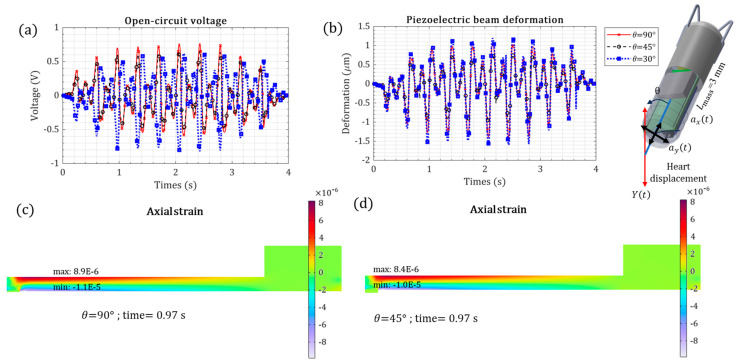
The piezoelectric cantilevered beam’s mechanical response under heart kinetic motion with different orientations of the intracardiac leadless pacemaker; (**a**) open-circuit piezoelectric voltage; (**b**) piezoelectric beam tip deformation; (**c**) axial strain with θ = 90° for t = 0.97 s, where the voltage is maximum; and (**d**) axial strain with θ = 45°mm for t = 0.97 s, where the voltage is maximum.

The orientation of intracardiac leadless pacemakers inside the right ventricle can differ, so that the heart’s cyclic displacement will differ. The effect of the orientation of the leadless pacemaker was studied, as shown in Figure 9. The angle of *θ* is between the axis of heart displacement motion and the leadless pacemaker’s central line. The piezoelectric beam’s vertical ($a_{y})$ and axial ($a_{x})$ base excitations can be estimated from the heart displacement (Y), as expressed by Equation (1).

(1) $$a_{y}=Y\left(t\right)\cos\theta \;;\;a_{x}=Y\left(t\right)\sin\theta$$

The vertical base excitation creates bending and thus contributes substantially to the energy generation compared to the axial excitation. The negative and positive *θ* have the same effect.

The peak voltage values were 0.76 V, 0.65 V, and 0.81 V for *θ* = 90°, 45°, and 30°, respectively, meaning a variation of 11%. Even though this variation can be considerable, it guarantees the maximum variation in energy generation with the pacemaker’s orientation.

The electrical performance was studied with purely resistive loads to test the power generation. The power obtained by the resistance load of 10 kΩ was 464.0 nW, with 6.8 μA current and 67.29 mV voltage.

A simple energy harvester beam model and sensitivity analysis demonstrated the potential of heart kinetic motion in energy harvesting. The real-time in vivo heart cardiac cycle was applied to a piezoelectric beam. Modifying structural and material configurations can be challenging and can substantially increase the output energy—this can be overcome by techniques such as tapered geometry and composite materials with fiber-variable direction [53] and variable-thickness piezoelectric layers [52].

**Figure 10:**
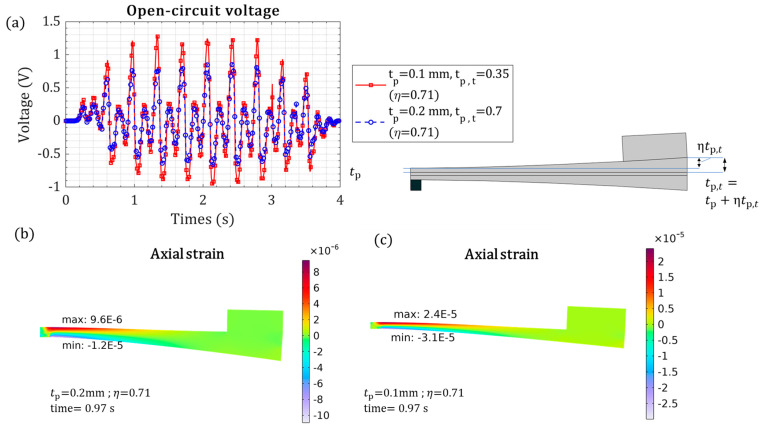
The investigation of variable-thickness piezoelectric layers, (**a**) open-circuit voltage for two parameters with different thickness, (**b**) axial strain for $t_{p}$ = 0.2 mm and $t_{p,t}$ = 0.7 mm (η = 0.71), and (**c**) axial strain for $t_{p}$ = 0.1 mm and $t_{p,t}$ = 0.35 mm (η = 0.71).

The performance of the energy harvester can be improved by variable thickness, as shown in Figure 10. The variable thickness effect of the piezoelectric layer on the axial strain and output voltage is demonstrated in Figure 10. Even though the thinner piezoelectric layer has less material volume, it generated better voltage output, which is due to higher axial strain.

Table 3 compares the different analyses of the piezoelectric energy harvester. As can be seen, the tip mass can increase the voltage by 36.6%, and the variable piezoelectric configuration can increase the voltage by 56.1%. The combination of these two parameters can increase the output voltage considerably. Other improvement features can be incorporated for better performance. On the other hand, the orientation of the piezoelectric energy harvester inside the heart can reduce the output voltage by −20.7%. This comparison shows that future heart energy-harvesting studies shall consider the energy harvester’s orientation.

**Figure 11:**
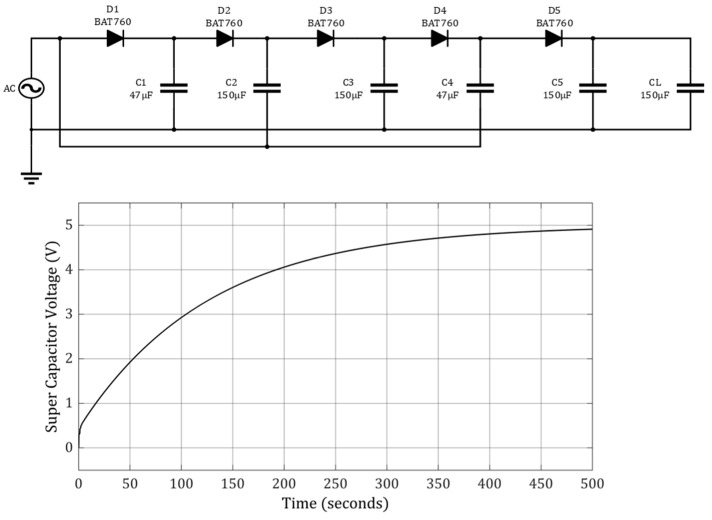
The electrical performance of a five-stage Dickson charge pump (MDCP) with an AC voltage source of 1.1 V and 1 Hz.

Electrical circuits are an important factor in energy harvesting. A piezoelectric energy harvester generates an alternative current (AC), which can be converted to a positive output through a typical bridge rectifier. The bridge rectifier has approximately 0.3 V loss [54]. Authors have previously investigated the multi-stage Dickson charge pump (MDCP) and shown the superior performance of the five-stage MDCP [55]. This five-stage MDCP was applied to the output of this energy harvester, as shown in Figure 11. After the five/stage MDCP, a 2-mF capacitor was used for the final energy storage. As shown in Figure 11, the output capacitor voltage reached approximately 5 V in 500 s. This simulation triggered advanced electrical circuits to increase the harvester output considerably. More studies are needed in this area.

## 4. Conclusions

The prospect of piezoelectric energy generation by the heart’s kinetic motion may lead to the self-powering of leadless pacemakers. The investigated prospects included the kinetic motion of a pig’s heart in an animal surgery trial test and electromechanical finite element analysis of piezoelectric energy harvesters. Heart motion has a high-level shock-inducing motion, leading to the main piezoelectric power generation and a low-level sinusoidal motion. A cantilevered beam was reported, and its electrical and mechanical performance were investigated. Different sensitivity studies were carried out, pointing out the most influential parameters. This work represents a starting point for energy harvesting with piezoelectric beams. The heart kinetic measurements were along the long axis of the heart only. This study presented a linear model and carried out improvement techniques for better performance. Despite the model’s simplicity, the aim was to demonstrate the practical potential of heart kinetic motion rather than relying on harmonic analysis of cardiac cycle motions, which is often the case in the literature. This study also emphasized that the harvester’s orientation inside the heart is important. In addition, variable piezoelectric thickness was shown to increase the output, and the multi-stage Dickson charge pump (MDCP) showed great potential in electrical management. This study’s analysis was limited to linear base excitation designs under one-dimensional heart excitation. Piezoceramic bulk materials were simulated.

More accurate studies should be conducted using three-dimensional heart motions and considering rotational motions. For further work, our team is developing a 3D axis accelerometer to be implanted inside the heart for additional measurements. Our team is currently exploring piezoelectric energy harvesting for self-powering medical devices. Power analysis using different resistance loads, power optimization under stopper conditions, and experimental studies are proposed as future works.

## Figures and Tables

**Table 1:** The properties of the quick pack bimorph piezoelectric harvester [52].

Description	Piezoelectric Sheets	Supporting Substrate	Bonding Layers
Material	PZT-5A	Brass	Epoxy
Length (mm)	L=57.2		
Width (mm)	b=31.8		
Thickness (mm)	$h_{p}$=0.19 (each layer)	$h_{s}$ =0.13	$h_{b}=0.02$ (each layer)
$\mathrm{Density}\;(\mathrm{kg}/m^{3}$)	$\mu_{p}=7870$	$\mu_{s}=8300$	$\mu_{b}=1400$
Elastic modulus in open circuit (GPa)	$Q_{11}^{E}$ $=Q_{22}^{E}$ =72	E*1s*=100	E*1b*=1.05
	$Q_{33}^{E}$ =100		
	$Q_{12}^{E}$ $=Q_{13}^{E}$ $=Q_{23}^{E}$ =22.3		
Poisson’s ratio	0.32	0.32	0.3
$\mathrm{Piezoelectric}\;\mathrm{coefficients}\;(C/m^{2}$)	$e_{31}$ $=e_{32}$ =−21.5 $e_{33}$ =61.8 $e_{15}$ =24.8	-	-
Relative permittivity	3800	-	-

**Table 2:** The first natural frequency between the experiments and the FE model.

Impact Force	The Open-Circuit First Natural Frequency (Hz)		Difference
	Experiment	COMSOL FE Model	
0.5 N	79.4	81.6	2.7%

**Table 3:** Comparison between different configurations of piezoelectric energy harvesters.

Variable Parameter Description	Variable Parameter [Best]	Fixed Parameters	Open Circuit Voltage	Difference with Default Configuration (%)
Tip mass weight	$L_{\mathrm{mass}}$ [= 6 mm]	$t_{p}$=0.2 mm θ=0° Constant thickness (η=0)	1.12 V	+36.6%
Piezoelectric layer thickness(default configuration)	$t_{p}$ [= 0.2 mm]	$L_{\mathrm{mass}}$=3 mm θ=0° Constant thickness (η=0)	0.82 V	-
Orientation of energy harvester inside the heart	$\theta \;\left[=0{}^{\circ}\right]$	$t_{p}$=0.2 mm $L_{\mathrm{mass}}$=3 mm Constant thickness (η=0)	θ=0° → 0.82 V θ=30° → 0.82 V θ=45° → 0.65 V θ=90° → 0.76 V	θ=0° → - θ=30° → 0% θ=45° →−20.7% θ=90° → −7.3%
Variable piezoelectric layer thickness	$t_{p}$ [=0.1 mm, η=0.71]	$t_{p}$ =0.2 mm $L_{\mathrm{mass}}$ =3 mm θ=0°	1.28 V	56.1%

## Data Availability

The original contributions presented in the study are included in the article, further inquiries can be directed to the corresponding author.
